# Differential Fatty Acid Response of Resident Macrophages in Human Skeletal Muscle Fiber and Intermuscular Adipose Tissue

**DOI:** 10.3390/ijms251910722

**Published:** 2024-10-05

**Authors:** Xiaoying Chen, Aline Müller, Miguel Pishnamaz, Frank Hildebrand, Leo Cornelius Bollheimer, Mahtab Nourbakhsh

**Affiliations:** 1 Clinic for Geriatric Medicine, RWTH Aachen University Hospital, 52074 Aachen, Germany; xchen@ukaachen.de (X.C.); almueller@ukaachen.de (A.M.); cbollheimer@ukaachen.de (L.C.B.); 2 Clinic for Orthopedics, Trauma, and Reconstructive Surgery, RWTH Aachen University Hospital, 52074 Aachen, Germany; mpishnamaz@ukaachen.de (M.P.); fhildebrand@ukaachen.de (F.H.)

**Keywords:** human, skeletal muscle, tissue model, tissue-resident macrophages, adipocytes, fatty acids, chemokines, cytokines, mitochondria

## Abstract

Human skeletal muscle contains different types of tissues with skeletal muscle fibers (SMFs) and intermuscular adipose tissues (IMATs) as the main components. We maintained human skeletal muscle tissues from 12 study participants under native conditions in vitro for 11 days to investigate the dynamics of macrophages that reside in adjacent IMATs and SMFs simultaneously. The samples were subjected to immunohistochemical analysis for macrophage phenotyping and mitochondrial mass assessment before and after maintenance in vitro. Multiplex protein analysis was used to determine cytokine/chemokine expression in tissue extracts. The results revealed significant correlations between donor age or body mass index (BMI) and distinct phenotypes of resident macrophages in SMFs and IMATs. The dynamics of SMF- and IMAT-resident macrophages differed significantly in vitro and exhibited inverse correlations with chemokine/cytokine expression levels and mitochondrial activity. Moreover, the responses of macrophages to saturated and unsaturated fatty acids (FAs) differed substantially between SMFs and IMATs. These findings showed the functional diversity of phenotypically identical macrophages in adjacent niches. Thus, the currently available macrophage markers cannot capture the functional diversity of human tissue-resident macrophages. The model used in the present study may help elucidate how macrophages affect muscle homeostasis and disease in humans.

## 1. Introduction

Human skeletal muscle tissue plays important roles in supporting body posture and movement as well as regulating metabolism and homoeostasis [1]. The hierarchical structure of skeletal muscle comprises bundles of skeletal myofibers (SMFs), intermuscular adipose tissues (IMATs), connective tissue, the vasculature, and motor neurons. The coordinated actions of these components are critical for skeletal muscle function and adjustment to changing conditions. Moreover, each structure is associated with specific populations of immune and stem cells that regulate inflammation and tissue regeneration.

IMAT is a depot of adipocytes that gradually accumulates between myofibers as a result of increased obesity [2]. Emerging evidence shows that IMAT expansion is closely linked to increased risk for developing type 2 diabetes (T2D) and insulin resistance as well as to aging and neuromuscular diseases that lead to reduced muscle mass [3,4]. The abundance of IMAT-resident macrophages was found to be increased in obese individuals [5]. Coculture experiments in vitro have shown that fatty acids (FAs) secreted from adipocytes increase glucose metabolism in the mitochondria of in vitro-differentiated myotubes [6]. In fact, several physiological factors, including body energy levels, exogenous energy from food, and the level of oxidative stress can affect mitochondrial activity in skeletal muscle [7]. Moreover, adipose tissue can secrete many hormones, adipokines, lipids, and miRNAs that also act on adjacent skeletal muscle cells [8]. Adipokines represent a group of cytokines/chemokines that regulate important metabolic or inflammatory pathways in adipose tissue associated with obesity and obesity-related disorders [9]. Similarly, skeletal muscle fibers secrete myokines that are involved in the autocrine regulation of metabolism in muscles and in para- or endocrine regulation of other tissues, including adipose tissue [10]. The increasing number of adipokines and myokines exposes intriguing overlaps, including interleukin (IL)-6, monocyte chemoattractant protein-1 (MCP-1), and tumor necrosis factor (TNF) alpha.

Tissue-resident macrophages (TRMs) represent a heterogeneous population of self-renewing immune cells. They regulate tissue homeostasis by modifying their location, morphology, and properties in response to injuries and diseases [11,12]. The temporal plasticity of TRMs has traditionally been studied in animal models, predominantly in rodents. For example, studies in mice have demonstrated that TRMs predominantly originate from the yolk sac during early embryonic development [13]. A recent study reported that the mouse lung TRMs specifically express a discrete set of chemokines in response to bacterial infections [14]. Previous animal studies have suggested that the responses and activities of TRMs may depend on their tissue niche. In humans, however, investigating the development and temporal dynamics of TRMs presents critical challenges, including ethical concerns and a lack of experimental models. Consequently, the available information on human TRMs is currently limited mainly to findings from static studies of TRM populations in human samples. The temporal dynamics of human TRM populations within native tissue remain widely unknown.

Previous in vitro studies of human blood- or bone marrow-derived monocytes have categorized macrophages in proinflammatory M1 and the anti-inflammatory M2 phenotypes [7]. This classification system advanced the identification of additional markers for macrophage detection and the development of corresponding antibodies for the study of macrophages in vitro [15]. However, emerging evidence has revealed that the M1/M2 classification may obscure a large spectrum of functionally relevant macrophages in vivo, especially TRMs [8]. A universal marker for TRMs is still lacking, and multiple genes expressed by monocyte-derived macrophages have been used for profiling TRMs and exploring their roles in disease and development [16]. Few markers have been verified in human pancreatic, lung, dermal, adipose, perivascular, and liver tissues. The most frequently studied human markers include CD11c, CD68, CD80, CD163, CD206, MARCO, and PTGER3.

The populations of SMFs- and IMATs-resident macrophages have not yet been thoroughly studied. A previously reported flow cytometry study of digested human skeletal tissue samples revealed the presence of CD206-expressing macrophages without specifying a particular niche [17]. A small number of bioengineered human skeletal muscle models have also been established for structural or functional studies of muscle fibers in disease or for drug discovery [18,19,20,21,22]. These models rely on 2D or 3D cultures of transformed human muscle cell lines, primary human myoblasts, or human-induced pluripotent stem cells. However, they lack important components, such as adipocytes and macrophages, and they cannot fully recapitulate the composite nature of native human skeletal muscle tissue. Recently, we established a procedure to maintain human skeletal muscle samples in vitro to investigate the actions of SMF stem cells and TRMs [23]. The present experimental study applied the same approach to compare the temporal alterations in TRM populations in adjacent SMFs and IMATs and in response to different species of FAs.

## 2. Results

### 2.1. The Abundance of Resident MARCO^+^, CD80^+^, PTGER3^+^ Macrophages in Human Skeletal Muscle Tissue Increases with Age and BMI

Human skeletal muscle tissue is typically composed of SMFs and IMATs. Herein, we aimed to investigate the different dynamics of SMF-resident TRMs and IMAT-resident TRMs in the native human tissue environment ex vivo simultaneously. The procurement of native human tissue samples is generally demanding due to ethical and logistical requirements. We were able to collect adequate amounts of skeletal muscle tissue specimens from the reconstruction surgeries of 12 adults in our hospital who provided informed consent for the donation of the disposed tissues. Most of the donors were female and older than 55 years. In addition, we included a 22-year-old female donor and two 65- and 67-year-old male donors. The body mass index (BMI) and diabetes type 2 status of the donors were determined before surgery. All donors were evaluated as medically fit and without pre-existing medical conditions before the surgeries. Table 1 summarizes the most relevant characteristics of all study participants.

**Figure 1:**
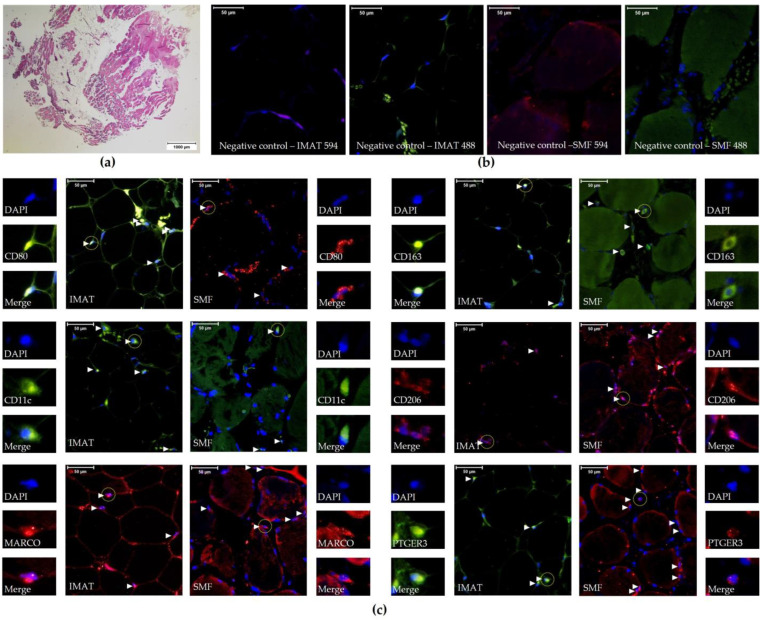
Representative images of skeletal muscle tissue samples. All images were obtained from participant P10. (**a**) The image of skeletal muscle tissue after HE staining indicating areas of SMFs and IMATs. (**b**) Immunofluorescence images of 230 µm × 260 µm fields of view after co-staining with DAPI and secondary antibodies as negative controls. (**c**) Immunofluorescence images of 230 µm × 260 µm fields of view after co-staining with primary antibodies against CD80, CD11c, MARCO, CD163, CD206, or PTGER3 with the corresponding secondary antibodies and DAPI. The small panels on the left show magnified single-cell images (dashed line circles) with DAPI (blue, top), IgG594 (red, middle), or IgG488 (green, middle) filters. DAPI and IgG594 or DAPI and IgG488 were merged (Merge, bottom) to determine the specificity of the detected signals. Verified positive cells are labeled with white arrowheads.

All samples were collected immediately after surgery and subjected to HE staining to distinguish SMFs and IMATs (Figure 1a). Among 23 reported TRM markers from different human tissues, 17 were excluded because of unavailability or the low detection specificity of the antibodies. Ultimately, we used primary antibodies against six markers, namely, CD80, CD11c, MARCO, CD163, CD206, and PTGER3, combined with fluorescently labeled secondary antibodies, for the IF staining of human skeletal muscle tissue samples. Control experiments using secondary antibodies alone yielded no significant labeling signals in SMFs or IMATs (Figure 1b). Co-staining of all the samples with DAPI confirmed the specificity and location of the positive signals (Figure 1b,c).

**Figure 2:**
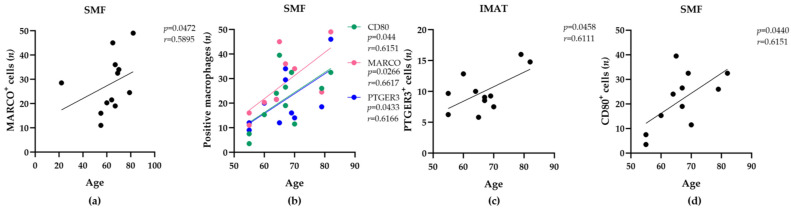
The abundance of tissue-resident macrophages is associated with donor age and BMI. Pearson correlation and Spearman’s rank correlation analyses were employed to identify statistically significant relationships between age (**a**–**c**) or BMI (**d**) and the number of macrophages of the indicated phenotypes (*y*-axis) within IMATs or SMFs (top of each diagram) among the donors (*n* = 12). The calculated correlation coefficients (r) and significance levels (*p*) are shown at the top right of each diagram. Differences with *p* ≤ 0.05 were considered statistically significant.

We determined the number of macrophages positive for each marker that were attached to SMFs or IMATs within two microscopic fields of view (0.24 mm^2^). The number of macrophage phenotypes (CD11c^+^, CD80^+^, CD163^+^, CD206^+^, MARCO^+^, or PTGER3^+^) between the SMFs or IMATs of the participants varied substantially (36- or 9-fold between SMFs and IMATs, respectively). However, we detected significant correlations between donor age and the abundance of SMF-resident or IMAT-resident CD80^+^, MARCO^+^, or PTGER3^+^ macrophages by further statistical analyses (Figure 2a–c). For example, the number of SMF-resident MARCO^+^ macrophages was significantly correlated with the age of the donors (Figure 2a, r = 0.59, *p* < 0.05). Among the participants older than 55 years, age was also correlated with increasing numbers of SMF-resident CD80^+^ (r = 0.62, *p* < 0.05) or PTGER3^+^ (r = 0.62, *p* < 0.05) macrophages (Figure 2b) and IMAT-resident PTGER3^+^ macrophages (r = 0.61, *p* < 0.05) (Figure 2c). These positive correlations suggested a general increase in the abundance of MARCO^+^, CD80^+^, and PTGER3^+^ macrophages with age. Moreover, independent of donor age, body mass index (BMI) was positively correlated with the number of SMF-resident PTGER3^+^ macrophages (Figure 2d, r = 0.68, *p* < 0.05). These findings suggest that the number of PTGER3^+^ macrophages may rise in human skeletal muscle with increasing age and BMI.

### 2.2. Macrophage Populations within Adjacent SMF and IMAT Differ

**Figure 3:**
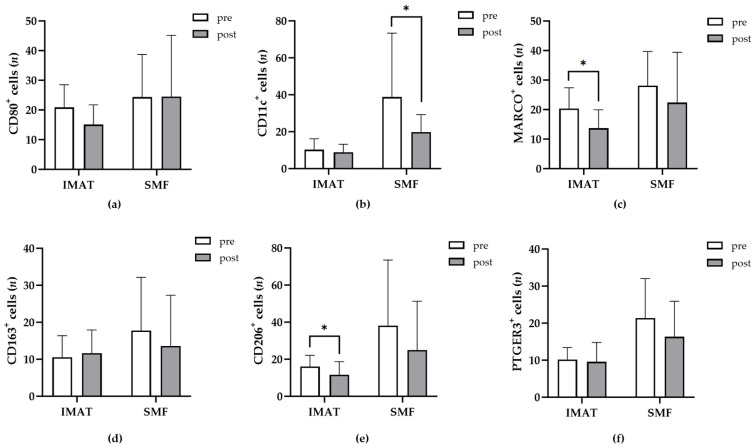
Macrophages of the same phenotype exhibit different trends in IMATs and SMFs. (**a**–**f**) The diagrams show the number of positive cells (*y*-axis) for different phenotypes (top of each diagram) in IMAT and SMF fields of 0.24 mm^2 ^(*x*-axis) in donors’ tissue samples (*n* = 12) before (white bars, pre) and after (gray bars, post) being maintained in vitro for 9 or 11 days. An unpaired *t* test or the Mann–Whitney test was employed to assess the significance of differences before and after cultivation. *p* ≤ 0.05 (*).

Numerous studies have suggested that TRMs can undergo proliferative and functional changes in animal models [24]. Here, we used a native human skeletal muscle tissue model to compare the temporal variations in SMF- and IMAT-resident macrophage populations in vitro. The donor tissue samples were maintained in vitro for 9 or 11 days and analyzed as described above (2.3.). We determined the number of CD80^+^, CD11c^+^, MARCO^+^, CD163^+^, CD206^+^, or PTGER3^+^ macrophages in SMFs and IMATs from all samples after 9 or 11 days (post) and compared them to the corresponding numbers that were obtained immediately after surgery (pre) (Figure 3a–f, respectively). Statistical analysis revealed no significant correlations between donors’ characteristics such as age or BMI and the changes in the abundance of phenotypes among IMATs or SMFs. Also, the number of CD80^+^, CD163^+^, or PTGER3^+^ TRMs in SMFs and IMATs did not change significantly (Figure 3a,d,f).

However, we observed significant changes in the number of SMF-resident CD11c^+^ and IMAT-resident MARCO^+^ or CD206^+^ macrophages (Figure 3b,c,e). Conversely, neither the number of IMAT-resident CD11c^+^ macrophages nor the number of SMF-resident MARCO^+^ or CD206^+^ macrophages were changed during the time of maintenance in vitro (Figure 3b,c,e). These observations demonstrated the different dynamics of CD11c^+^, MARCO^+^ and CD206^+^ macrophages in adjacent SMFs and IMATs. Thus, macrophages positive for the same marker may act differently when resident in adjacent tissue of different types.

### 2.3. Alterations in the Abundance of the IMAT- and SMF-Resident Macrophage Populations Are Correlated with Selective Expression of Cytokines/Chemokines

**Figure 4:**
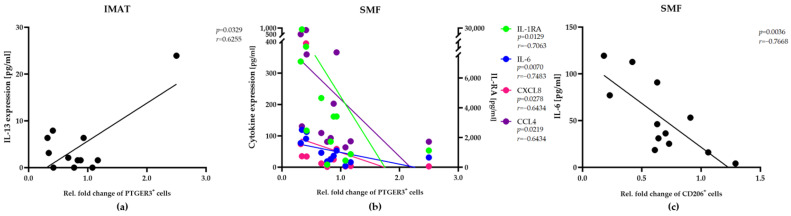
Different sets of cytokines/chemokines regulate the number of PTGER3^+^ macrophages in adjacent SMFs and IMATs. Pearson correlation and Spearman’s rank correlation analyses were employed to determine the relationships between the relative fold changes in PTGER3^+^ (**a**,**b**) or CD206^+^ (**c**) macrophage numbers in IMATs (**a**) or SMFs (**b**,**c**) of the donors (*n* = 12) and the tissue expression levels of different cytokines/chemokines (*y*-axis). The correlation coefficients (r) and significance levels (*p*) for the relationships are presented on the top right or left side of the diagrams.

Next, we analyzed the expression of multiple cytokines/chemokines to evaluate the possible link between the levels of inflammatory proteins and the relative fold changes in the abundance of specific phenotypes among SMF- or IMAT-resident macrophage. To obtain the relative fold changes, we normalized the numbers of each phenotype in SMFs and IMATs after maintenance to their corresponding numbers immediately after surgery. Thus, values higher or lower than one signify increases or decreases in macrophage abundance, respectively (Figure 4a,b, *x*-axis). Remarkably, we observed a positive correlation between the expression level of IL-13 and the relative fold increase in IMAT-resident PTGER3^+^ macrophages (Figure 4a). In SMFs, however, the expression levels of four chemokines, IL-1RA, IL-6, IL-8 and MIP-1 beta, were correlated with decreases in the number of PTGER3^+^ macrophages (Figure 4b). These findings suggested that the number of PTGER3^+^ macrophages in adjacent SMFs and IMATs may be regulated by different sets of chemokines. IL-6 levels were correlated with a decrease in the number of SMF-resident CD206^+^ macrophages in our samples (Figure 4c). However, we detected no significant correlations with other cytokines/chemokines (Supplementary Materials, Table S1).

### 2.4. Alterations in the Abundance of IMAT- and SMF-Resident Macrophage Populations Are Correlated with Mitochondrial Activity

**Figure 5:**
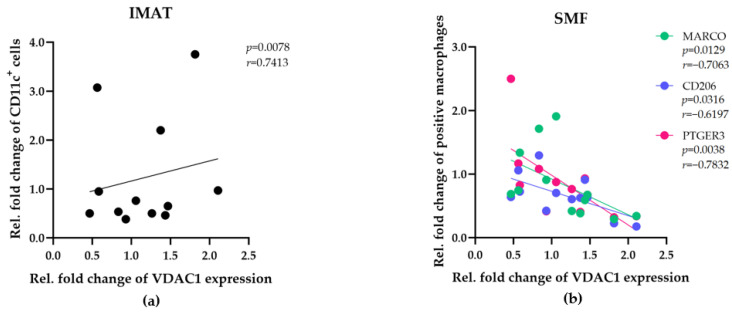
The abundance of resident macrophages in IMATs and SMFs correlates with the mitochondrial mass. Pearson correlation and Spearman’s rank correlation analyses were employed to determine the relationships between VDAC1 expression levels and the fold change in the number of indicated TRMs in IMATs (**a**) and SMFs (**b**) of the donors (*n* = 12). The correlation coefficients (r) and significance levels (*p*) for the relationships are presented on the top right of each diagram.

The VDAC1 expression level is linked to mitochondrial mass in skeletal muscle fibers and adipose tissues [25,26]. To compare the means of metabolic activities during the tissue maintenance in vitro, VDAC1 levels in SMFs and IMATs were determined and normalized to the number of muscle fibers or adipocytes, respectively. VDAC1 was significantly higher expressed in SMFs than in IMATs, but its expression in IMAT was increased during the tissue maintenance in vitro (Supplementary Materials, Figure S1). Statistical analyses revealed opposite correlations between VDAC1 expression and the relative fold changes in IMAT and SMF macrophage numbers in vitro (Figure 5a,b, Supplementary Materials, Table S2). In IMATs, increasing numbers of CD11c^+^ macrophages were positively correlated (*p* = 0.0078, *r* = 0.7413) with VDAC1 expression (Figure 5a). In SMFs, increasing numbers of MARCO^+^, CD206^+^, and PTGER3^+^ macrophages were negatively correlated (*p* = 0.0129, *r* = −0.7063, *p* = 0.0316, *r *= −0.6197, and *p* = 0.0038, *r* = −0.7832, respectively) with VDAC1 expression (Figure 5b). Thus, the mitochondrial mass in IMATs and SMFs may contribute to the abundance of resident macrophages.

### 2.5. The Increase in Macrophage Populations in Response to FAs Is Significantly Different between IMATs and SMFs

**Figure 6:**
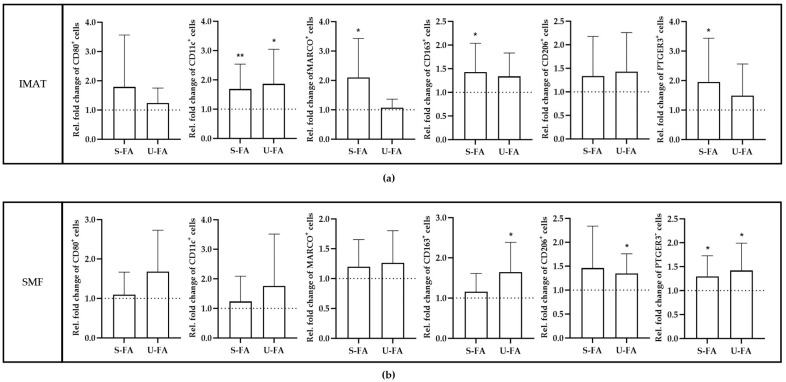
FAs exert disparate effects on SMF- and IMAT-resident macrophages. Adjacent tissue samples from all donors (*n* = 12) were maintained in vitro for 9 or 11 days with or without U-FAs (C16[1]c and C18[2]c) or S-FAs (C16 and C18). The number of cells in FA-stimulated tissues was normalized to the corresponding number of cells in untreated tissues to calculate the relative fold change in response to S-FAs or U-FAs. For comparison, the relative fold change in untreated tissues was set to 1 (dashed line). The diagrams show the relative fold changes in cell numbers (*y*-axis) for the specified macrophage phenotypes (top of each diagram) after treatment with S-FAs or U-FAs (*x*-axis) in IMATs (**a**) or SMFs (**b**) of the donors (*n* = 12). One-sample *t* tests or the Wilcoxon signed-rank test was employed to assess the significance of the detected differences. *p* ≤ 0.05 (*), *p* ≤ 0.01 (**).

Previous studies have suggested that elevated levels of fatty acids can induce localized tissue inflammation via the selective accumulation of proinflammatory resident macrophages [27]. However, the role of the target tissue and the profile of resident macrophages remained unclear. We hypothesized that SMF- and IMAT-resident macrophages may respond differently to external stimuli such as fatty acids (FAs). Therefore, a series of tissue sections from all participants were maintained in vitro with or without FAs for 9 or 11 days. We used saturated C16 and C18 or unsaturated C16[1]c and C18[2]c. In these designations, the number of carbon atoms is depicted first, which is followed by the number of unsaturated carbon double bonds in square brackets. In addition, both unsaturated FAs, C16[1]c and C18[2]c, were in the cis configuration, as indicated by the letter “c”. We observed no significant differences between C16 and C18 or between C16[1]c and C18[2]c and presented the data as a summary of unsaturated FAs (U-FAs) or saturated FAs (S-FAs) (Figure 6a,b). In general, IMAT-resident macrophages (CD11c^+^, MARCO^+^, CD163^+^, and PTGER3^+^) were increased by saturated FAs (Figure 4a), whereas SMF-resident macrophages (CD163^+^, CD206^+^, and PTGER3^+^) were primarily increased in response to unsaturated FAs (Figure 6b). Although the relative fold changes varied between the donors, the significance level of the differences between the samples was very high (Table 2). In comparison, the only shared response between SMFs and IMATs was the accumulation of PTGER3^+^ macrophages in response to S-FAs. Most importantly, macrophages of the same phenotype responded differently within SMFs and IMATs.

### 2.6. The FA-Induced Increase in Macrophage Populations Is Inversely Orchestrated between IMATs and SMFs

**Figure 7:**
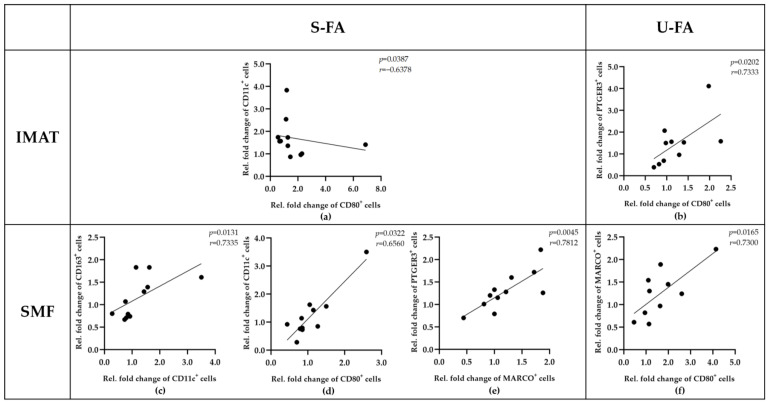
The accumulation of macrophages of different phenotypes in response to FA treatment markedly differed between IMATs and SMFs. Pearson correlation and Spearman’s rank correlation analyses were conducted to determine the relationships among the relative fold changes in abundance of macrophage phenotypes across all samples (*n* = 12) following stimulation with U-FAs or S-FAs (top of each diagram). The calculated correlation coefficients (r) and significance levels (*p*) are displayed in the diagrams. (**a**,**b**) Correlations between the relative fold changes in the abundance of macrophage phenotypes (*x*-axis and *y*-axis) in IMATs following treatment with U-FAs or S-FAs. (**c**–**f**) Correlations between the relative fold changes in the abundance of macrophage phenotypes (*x*-axis and *y*-axis) in SMFs after treatment with U-FAs or S-FAs. Differences for which *p* was ≤0.05 were considered statistically significant.

The results in Figure 6a,b revealed that S-FAs and U-FAs can equally increase the numbers of IMAT-resident CD11c^+^ macrophages and SMF-resident PTGER3^+^ macrophages. This prompted us to investigate whether the responses of distinct macrophage phenotypes are distinctly coordinated in SMFs and IMATs. Therefore, we examined potential correlations between the number of different macrophage phenotypes in response to S-FAs and U-FAs (Figure 7). In IMATs, the increase in CD80^+^ macrophages was negatively correlated with CD11c^+^ macrophages in response to S-FA (*p* = 0.0387, *r* = −0.6378) but positively correlated with PTGER3^+^ macrophages in response to U-FA (*p* = 0.0202, r = 0.7333) (Figure 7a,b). These results indicated that S-FAs and U-FAs can differentially affect the macrophage population in IMATs. Most importantly, however, S-FAs can exert apposite effects on IMATs and SMFs (Figure 7a,d). The S-FA mediated increases in CD80^+^ and CD11c^+^ macrophages were positively correlated in SMFs (*p* = 0.0322, *r* = 0.6560) but negatively in IMATs (*p* = 0.0387, *r* = −0.6378) (Figure 7a,d). Together, these results highlight the importance of tissue residence for the dynamic responses of resident macrophages independent of their marker profile.

## 3. Discussion

Elucidating the functional diversity of TRMs requires temporal monitoring of their phenotype-specific activities under experimentally controlled changing conditions. This is not feasible in humans because of ethical concerns. The present study used human skeletal muscle tissue ex vivo and was the first attempt to examine the spatiotemporal plasticity of human skeletal muscle TRMs under experimental conditions. Our differential analysis of IMAT- and SMF-resident macrophages provided evidence that the variability of TRM populations is regulated by their surrounding tissues even when they are in spatial proximity. Notably, the distance between the analyzed IMATs and SMFs in our study ranged from 10 to 120 µm. The observed differences between the IMAT- and SMF-resident macrophage phenotypes were substantiated by six well-established TRM phenotypic markers. All the phenotypes exhibited disparate dynamics depending on cytokine/chemokine levels, FA species, and their location in IMATs and SMFs. Although these markers alone are unlikely to reveal tissue-specific TRM functions, the spatiotemporal study of human composite tissues via advanced technologies may reveal functional markers that can gradually reveal the influence of the tissue microenvironment and the functional characteristics of human TRMs.

Our ex vivo model of human skeletal muscle tissue provided valuable insights into the dynamics of TRM populations under controlled conditions. The maintenance conditions had no significant effects on the mean number of CD80^+^, CD163^+^, or PTGER3^+^ TRMs (Figure 3a,d,f). Other phenotypes (CD11c^+^, MARCO^+^, and CD206^+^) were declined either in SMFs or in IMATs exclusively (Figure 3b,c,e). These selective trends are unlikely due to the general impairment of cell viability in vitro. Moreover, we did not detect any signs of declining cell viability such as stronger DAPI signal or altered nuclear morphology in tissue samples that were maintained in vitro for 9 or 11 days in the present or previous studies [23,28]. Nevertheless, the lifespan of our ex vivo model may be limited to only 11 days, which is longer than the reported lifespan of other musculoskeletal in vitro models [29].

Previous clinical studies have associated IMAT expansion with the critical loss of skeletal muscle mass and strength that is defined as sarcopenia [3,4]. A more direct correlation between IMAT expansion and muscle mass decline was concluded from clinical studies on patients with sarcopenic obesity (SO), which is characterized by the cooccurrence of obesity and sarcopenia [30,31]. Moreover, IMAT expansion was significantly correlated with the increased abundance of IMAT-resident macrophages and elevated inflammatory factors in SO patients [5,32]. However, the SMF-resident TRMs in SO patients were not further studied yet. This may be due to the general challenges of attaining adequate human specimens, which was also a limitation of our study model. Although our study did not involve SO patients, the outcomes provided evidence for the differential dynamics of TRM populations in SMFs and IMATs as well as in response to FAs that may be relevant to the field of SO. Therefore, it is tempting to speculate that the inclusion of SO muscle tissue in our ex vivo model may provide deep insights into the underlying regulatory crosstalk between IMAT and SMF that drives cellular processes in SO.

Inflammation has long been associated with the age-related loss of skeletal muscle mass and function. Many recent insights into the effects of age on inflammation in skeletal muscle were derived from cell culture experiments or animal models. Only a few studies have examined the inflammatory state of macrophages that reside in the skeletal muscle tissue of older individuals [33,34]. These studies analyzed SMF-resident CD206^+^ or CD163^+^ macrophages and reported no significant differences between older (63–75 years) and younger adults (22–38 years) [33,34]. Accordingly, we found no correlations between the number of SMF-resident CD206^+^, CD163^+^, or CD11c^+^ macrophages and the participants’ age. Nevertheless, the abundance of SMF-resident CD80^+^, MARCO^+^, and PTGER3^+^ macrophages increased with age in our study, indicating a significant effect of aging on the inflammatory status of skeletal muscle tissue.

The role of macrophages in human diseases has been investigated by analyzing macrophage phenotypes in clinical samples. For instance, obesity has been often associated with the activation and infiltration of macrophages in abdominal adipose tissue and chronic low-grade inflammation [35]. Prolonged inflammation reduces the ability of adipocytes to store lipids, leading to increased lipid deposition in IMATs [36]. Moreover, IMAT typically expands between muscle fibers or adjacent muscle bundles in obese individuals [37,38]. A previous study reported an obesity-induced increase in PTGER3 expression in macrophages extracted from subcutaneous adipose tissue without the dissection of tissue components [39]. In our study, increasing BMI, a measure of obesity, was significantly associated with the accumulation of PTGER3^+^ macrophages in the SMFs but not IMATs of the participants. This led to the suggestion that PTGER3^+^ macrophages may play a different role in the obesity-induced expansion of subcutaneous adipose tissue than in IMAT.

Numerous studies on metabolic and inflammatory disorders in humans and animals have reported the regulatory effects of skeletal muscle tissue and TRMs [40]. In the present study, several observations suggest a more complex scenario that involves differential behaviors of SMF- and IMAT-resident macrophages. In general, IL-13 expression and mitochondrial mass were positively correlated with the number of IMAT-resident PTGER3^+^ and CD11c^+^ macrophages, respectively. Indeed, IL-13 has been reported to exert autocrine effects in skeletal muscle to promote metabolism [41]. In SMFs, however, chemokine/cytokine expression and mitochondrial mass were negatively correlated with the number of PTGER3^+^ macrophages. Thus, it is tempting to speculate that PTGER3^+^ macrophages exert opposite inflammatory and metabolic effects on IMATs and SMFs.

Obesity has been associated with the dysregulation of adipose tissue, which undergoes basal lipolysis and FA leakage, causing local inflammation [27]. In agreement with these findings, our data demonstrated that FAs could increase the number of macrophages of most phenotypes, including the CD11c^+^, MARCO^+^, CD163^+^, CD206^+^, and PTGER3^+^ phenotypes, in skeletal muscle tissues; however, these effects depend on the type of FA or the tissue niche. Remarkably, an increase in the number of CD163^+^ macrophages was observed in SMFs specifically in response to U-FA treatment, whereas an increase in the number of S-FAs was observed in IMATs. A recent study of human visceral adipose tissue samples revealed that the number of CD163^+^ macrophages positively correlated with the concentration of U-FAs (linolenic acid) in the tissue [42]. Thus, CD163^+^ macrophages may perform different functions depending on their surrounding tissue niche, as suggested for PTGER3^+^ macrophages. Moreover, our data suggest that FAs may affect the coordinated actions of two different TRM phenotypes in a specific tissue niche. For example, the increases in the numbers of CD163^+^ and CD11c^+^ macrophages in SMFs were significantly correlated with the response to S-FAs (Figure 7).

Skeletal muscle tissue from obese individuals or type 2 diabetes patients exhibits the accumulation of anti-inflammatory M2 macrophages on the basis of elevated expression of the CD163 and CD206 proteins [43,44]. Based on the disparities between the macrophage populations in SMFs and IMATs from human skeletal muscle tissues, as well as their selective responses to FAs in our study, we suggest that the role of skeletal muscle-resident macrophages in the development of human conditions, such as obesity, aging, and type 2 diabetes, may be more complex than can be explained by the restrictive M1/M2 paradigm. We found that variable combinations of M1 (CD80, MARCO, and CD11c) and M2 (CD163, CD206, and PTGER3) macrophages simultaneously accumulated in IMATs or SMFs with increasing age. Additionally, we observed significant differences in the numbers of CD206^+^ (*p* = 0.0035) and PTGER3^+^ (*p* = 0.0031) macrophages between SMFs and IMATs in this study. These differences were also observed for a participant with type 2 diabetes and a younger participant.

**Figure 8:**
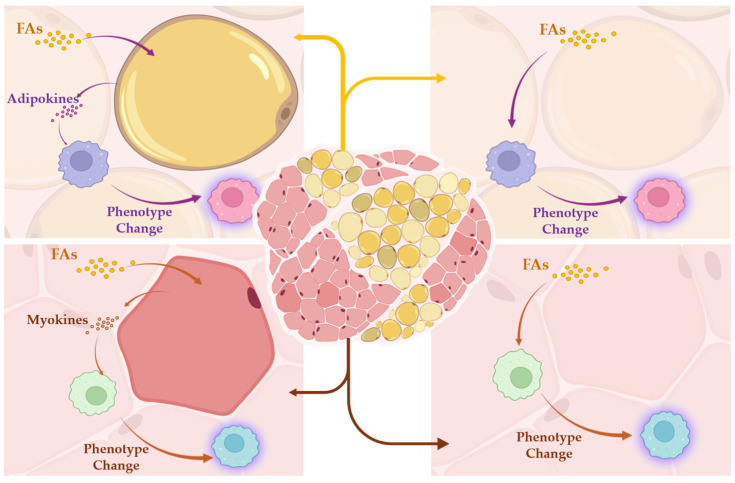
Schematic presentation of hypothetical direct (**right panels**) and indirect effects of FAs on TRMs phenotype change via activation of adipocytes or muscle fibers (**left panels**). IMATs (**upper panels**) and SMFs (**lower panels**) are shown including adipocytes and muscle fibers, respectively.

The role of TRMs in the skeletal muscle mass loss associated with age, SO or type 2 diabetes needs to be verified in larger groups of participants, which was a limitation of our study model. Another limitation of our study was due to the relatively long timescales of observations ex vivo. Future serial studies will be necessary to elucidate the dynamics of FAs-stimulated signal transductions and their immediate effects on TRMs in SFM and IMATs simultaneously. The outcomes will reveal whether different FAs can exert direct and/or indirect effects on TRMs via the activation of adipocytes or muscle fibers as depicted in Figure 8. Lastly, the outcomes of our study were limited to six commonly known detection markers for human macrophages. However, ours and other observations strongly imply that the current markers cannot reflect the diversity of TRM functions. Thus, future analysis of our study model using spatial transcriptomics can accelerate the identification of functional markers for human skeletal muscle TRMs.

## 4. Materials and Methods

### 4.1. Collecting of Skeletal Muscle Tissue Specimens

Tissue specimens were acquired from patients (*n* = 12) who were surgically treated in the Clinic for Orthopedics, Trauma, and Reconstructive Surgery at RWTH Aachen University Hospital in Aachen, Germany. The excised tissue was placed in a sterile container by the surgeon and sent to the lab immediately. The present study was approved by the Medical Ethics Committee of RWTH Aachen University before sample collection (No. EK206/09). All participants and surgeons have signed informed consent documents that are retained in the Centralized Biomaterial Bank (cBMB) at RWTH Aachen University Hospital in Aachen, Germany.

### 4.2. Quantification of Cytokines/Chemokines Expression in Tissue Samples

Preconfigured ProcartaPlex assays (EPXR340-12167-901, Thermo Fisher Scientific, Waltham, MA, USA) were used to prepare tissue extracts from 2.5 mg of tissue and quantify the concentration of 34 human cytokines/chemokines (Eotaxin (CCL11), granulocyte macrophage colony-stimulating factor (GM-CSF), growth-regulated protein (GRO) alpha (CXCL1), interferon (IFN) alpha, IFN gamma, IL-1 alpha, IL-1 beta, IL-10, IL-12p70, IL-13, IL-15, IL-17A (CTLA-8), IL-18, IL-1RA, IL-2, IL-21, IL-22, IL-23, IL-27, IL-31, IL-4, IL-5, IL-6, IL-7, IL-8 (CXCL8), IL-9, IFN gamma-induced protein (IP)-10 (CXCL10), MCP-1 (CCL2), macrophage inflammatory protein (MIP)-1 alpha (CCL3), MIP-1 beta (CCL4), regulated on activation, normal T-cell expressed and secreted (RANTES) (CCL5), stromal cell-derived factor (SDF)-1 alpha, TNF alpha, TNF beta), according to the manufacturer’s instructions. The mean of four independent reads has been used as the final concentration and statistical analysis.

### 4.3. Handling and Maintaining of Human Tissue Samples

All the obtained tissue samples were evenly cut into 18 mm^3^ sections. As an initial reference, a single section was treated with 4% formaldehyde for 24 h (Otto Fischar GmbH, Saarbrucken, Germany) immediately after dissection. The remaining sections were individually embedded in a gel medium composed of 1% low-melt agarose (Carl Roth GmbH, Karlsruhe, Germany), 10% fetal bovine serum (PAN Biotech GmbH, Aidenbach, Germany), 100 U/mL penicillin, and 100 U/mL streptomycin (PAN Biotech GmbH) in DMEM (Biological Industries, Kibbutz Beit-Haemek, Israel) at 40 °C. For FA treatments, FAs (FA 1208, 1024, 1014, and 1020, Biotrend Chemikalien GmbH, Cologne, Germany) were solved at a 1:2.5 ratio in 10% bovine serum albumin (BSA) in PBS and added to the gel medium at a final concentration of 50 μM. DMEM/F-12 medium (Gibco, Thermo Fisher, Waltham, MA, USA) containing 10% fetal bovine serum and 100 U/mL penicillin and streptomycin were added on the top of the gel medium after it was completely solidified at room temperature. The embedded tissues were maintained at 37 °C with 5% CO_2_. After 9 or 11 days, the supernatant was removed, and the tissue samples were carefully removed from gel medium and incubated in 4% formaldehyde solution (Otto Fischar GmbH) for 24 h.

### 4.4. Hematoxylin and Eosin (HE) Staining

Tissue samples were dehydrated in ascending alcohol concentrations (70, 96, and 100%) and embedded in paraffin blocks. A SLIDE4003E microtome (pfm Medical, Cologne, Germany) was used for the sectioning of the paraffin blocks. The tissue slices of 5 μm were then attached to glass slides. An automated slide-staining station (Gemini, Thermo Fisher, Waltham, MA, USA) was used to deparaffinize and stain with hematoxylin for 5–10 min, wash with warm water for 10 min, stain with 0.3% eosin for 5 min, and wash again with distilled water. The slides were dehydrated in ascending alcohol concentrations (70, 96, and 100%), treated with xylene, and sealed with glass cover slips.

### 4.5. Immunofluorescence (IF) Detection of Human Macrophage Markers

Paraffin tissue slides were deparaffinized and then heated in citrate buffer (pH 6.0) for 30 min. After cooling in water, the slides were washed twice in 0.1% Tween 20 (9127.1, Carl Roth) in PBS and blocked in UltraCruz Blocking Reagent (Santa Cruz Biotechnology, Dallas, TX, USA) for 60 min. Primary antibodies against human CD80 (1:1000, ab134120, Abcam, Cambridge, UK), CD11c (1:100, ab52632, Abcam), MARCO (1:100, PA5-64134, Thermo Fisher Scientific), CD163 (1:200, ab156769, Abcam), CD206 (1:100, PA5-101657, Thermo Fisher Scientific), PTGER3 (1:100, PA5-102057, Thermo Fisher Scientific), or VDAC1 (1:100, ab154856, Abcam) were diluted in 3% BSA in PBS as indicated. For staining, the slides were incubated with primary antibodies overnight at 4 °C; then, they were washed and incubated with secondary antibodies, anti-rabbit IgG488 (ab150081, Abcam), anti-rabbit IgG594 (ab150084, Abcam), or anti-mouse IgG488 (ab150117, Abcam), which was followed by being diluted 1:200 in UltraCruz Blocking Reagent for 60 min. After the wash, the slides were incubated in 0.1% DAPI (D9542, Sigma–Aldrich, St. Louis, MO, USA) in PBS for 5 min and then sealed with glass coverslips in Immu-Mount (9990402, Thermo Fisher Scientific).

### 4.6. HE and IF Imaging

An automated microscope (DM6000B, Leica Microsystems, Wetzlar, Germany) with an integrated camera was used for microscopy and obtaining HE and IF images. DAPI, anti-rabbit IgG488, or anti-mouse IgG594 images were obtained using 340–380 nm, 450–490 nm, or 590 nm filters, respectively. The Diskus software version 10 (Leica) was utilized to process and merge images. For each individually stained section, at least two random fields of view (0.24 mm^2^) in cross-sectional SMFs and IMATs were selected and evaluated to determine the number of positive cells. Throughout the evaluation process, we specifically analyzed SMFs and IMATs and strictly excluded all other areas of known or unknown function. ImageJ software v1.54g (https://imagej.net/, accessed on 11 July 2024) was used to process and evaluate VDAC1 images and expression, respectively.

### 4.7. Statistical Data Analysis

The normality of the data distribution was examined using the Shapiro–Wilk test. Pearson’s or Spearman’s rank correlation analysis was utilized to assess the correlations between cell numbers, cytokines/chemokines expression, or clinical characteristics. Changes in normalized values were evaluated via one-sample *t* tests or the Wilcoxon signed-rank test. Differences in cell counts between IMATs and SMFs, as well as between before and after tissue maintenance in vitro, were analyzed via an unpaired t-test or the Mann–Whitney test. GraphPad Prism (version 10.1.2, GraphPad Software, San Diego, CA, USA) was used for all statistical analyses. Differences at *p* values ≤ 0.05 were considered as statistically significant.

## 5. Conclusions

Our human ex vivo model can emphasize the diversity of macrophage populations that reside in two different tissue niches of human skeletal muscle. By performing a spatiotemporal comparison of six different macrophage phenotypes, we gained insight into the similarities and differences in their coordinated activities and responses to FAs. Future studies are required to examine whether FAs can affect macrophages directly and/or indirectly via the stimulation of adjacent adipocytes and muscle fibers that secrete adipokines and myokines, respectively (Figure 8). Moreover, the functional diversity of TRMs beyond the utilized markers remains to be further elucidated. The presented study model combined with advanced emerging techniques, such as spatial transcriptomics, may greatly improve our understanding of regulatory functions of TRMs in human diseases or the influence of the tissue microenvironment on TRM expression profiles in the future.

## Figures and Tables

**Table 1:** Participant characteristics.

Participants	Sex	Age (Years)	BMI (kg/m^2^)	Type 2 Diabetes (T2D)
P1	Female	79	22.8	No
P2	Female	67	46.9	Yes
P3	Female	55	16.7	No
P4	Female	64	33.8	No
P5	Female	60	22.1	No
P6	Female	55	22.6	No
P7	Female	69	22.3	No
P8	Female	70	20.0	No
P9	Female	82	30.5	No
P10	Male	67	28.6	No
P11	Female	22	20.3	No
P12	Male	65	24.2	No

**Table 2:** Significance levels of FA-mediated accumulation in IMAT-or SMF-resident macrophages.

	IMAT		SMF	
	S-FA	U-FA	S-FA	U-FA
**CD80**	0.1406	0.1684	0.1406	0.0710
**CD11c**	**0.0098**	**0.0273**	**0.0098**	0.1602
**MARCO**	**0.0210**	0.4209	**0.0210**	0.1598
**CD163**	**0.0427**	0.0592	**0.0427**	**0.0224**
**CD206**	0.4648	0.3613	0.4648	**0.0261**
**PTGER3**	**0.0244**	0.1934	**0.0244**	**0.0195**

The significance levels *p* ≤ 0.05 are highlighted in bold.

## Data Availability

The data presented in this study are available in Supplementary Materials, available online at www.mdpi.com.

## Supplementary Materials

The supporting information can be downloaded at https://www.mdpi.com/article/10.3390/ijms251910722/s1.
