# Supplementary figures and images for: Differential Fatty Acid Response of Resident Macrophages in Human Skeletal Muscle Fiber and Intermuscular Adipose Tissue
Source: Int J Mol Sci. 2024 Oct 5;25(19):10722. doi: 10.3390/ijms251910722 (PMC11477279; doi:10.3390/ijms251910722)

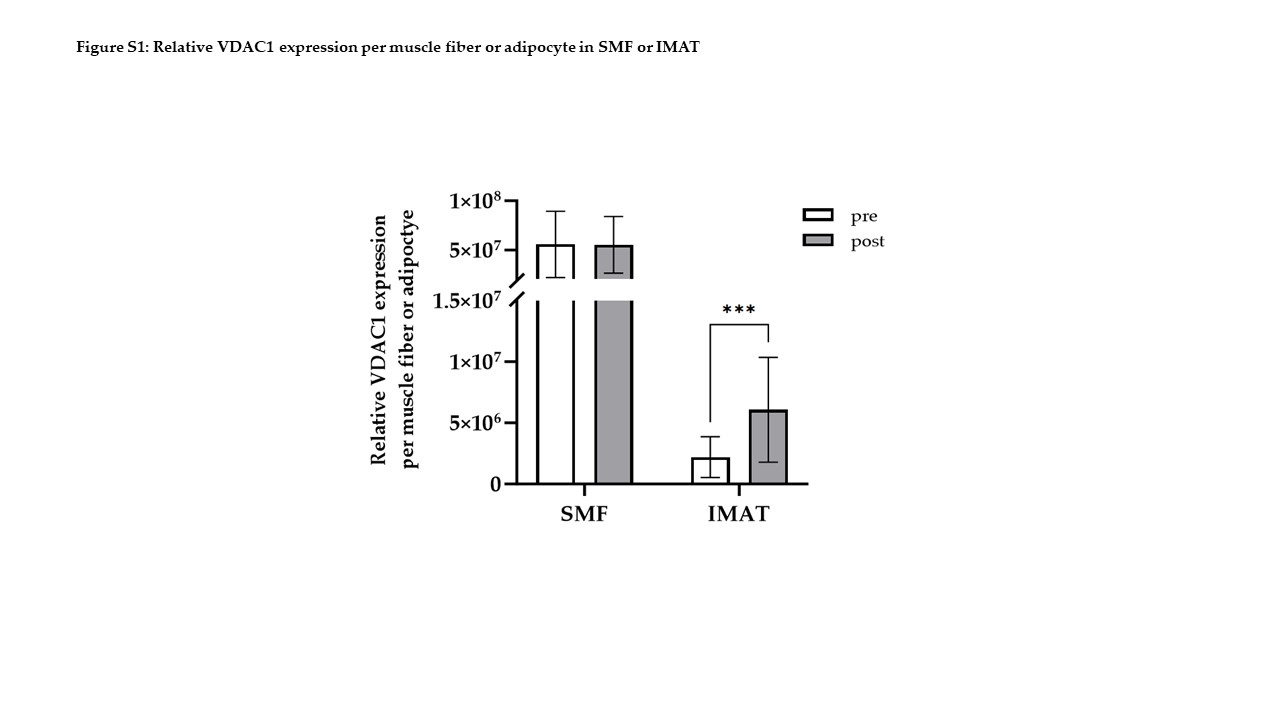

Supplement: Supplementary file 1 [file ijms-25-10722-s001.zip › FigS1.JPG]

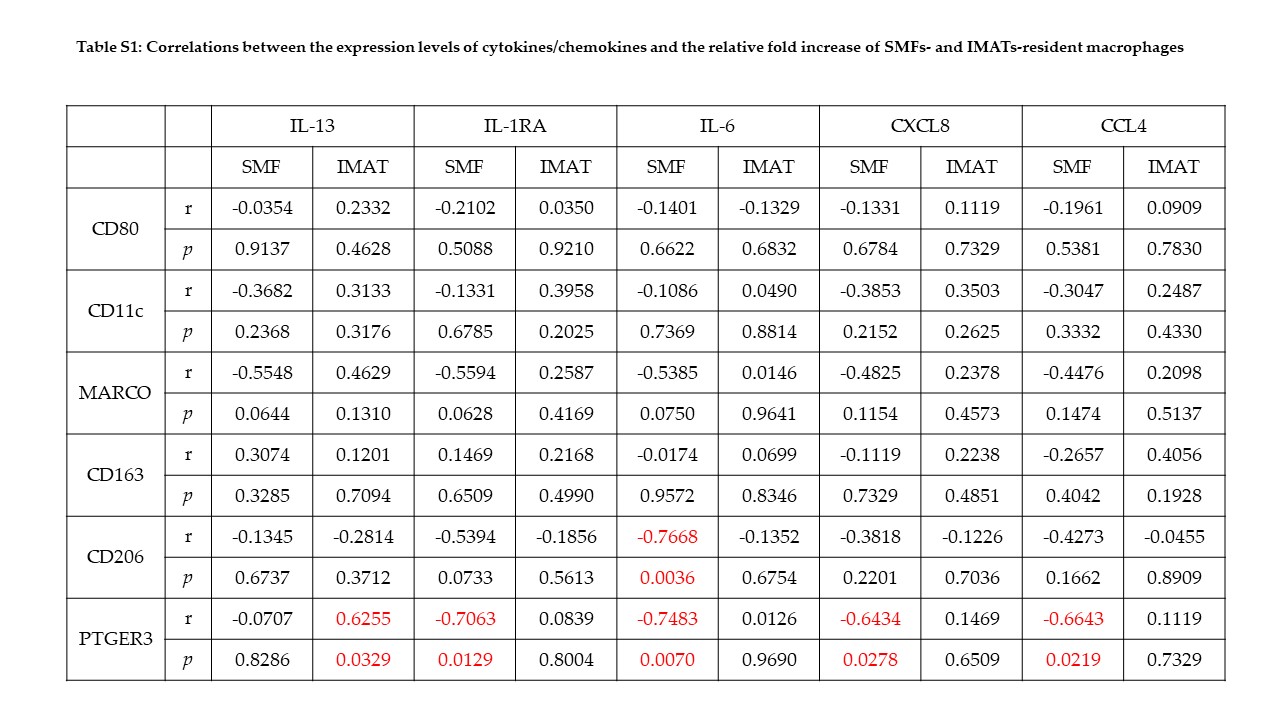

Supplement: Supplementary file 1 [file ijms-25-10722-s001.zip › TabS1.JPG]

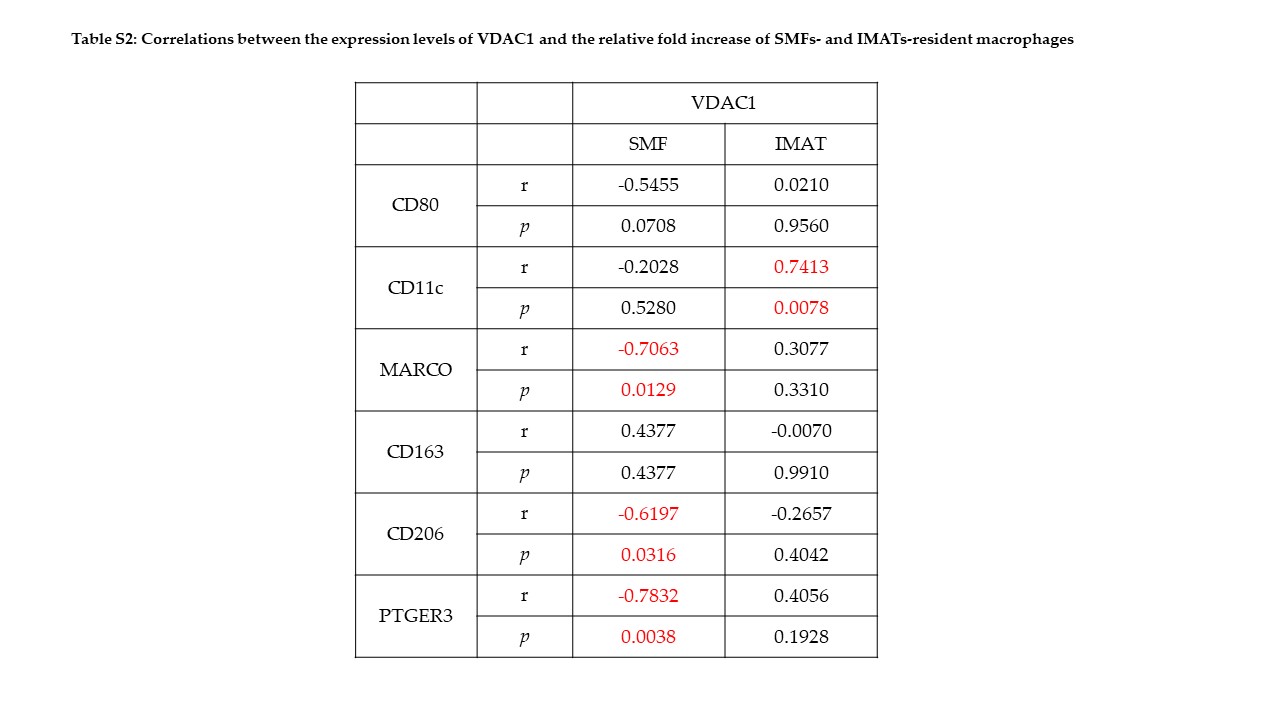

Supplement: Supplementary file 1 [file ijms-25-10722-s001.zip › TabS2.JPG]
